# A review of the LARIAT device: insights from the cumulative clinical experience

**DOI:** 10.1186/s40064-015-1289-8

**Published:** 2015-09-17

**Authors:** Mukta C. Srivastava, Vincent Y. See, Murtaza Y. Dawood, Matthew J. Price

**Affiliations:** University of Maryland School of Medicine, 110 S. Paca Street; 7N-121, Baltimore, MD 21201 USA; Scripps Clinic, 10666 N. Torrey Pines Road, La Jolla, CA USA

**Keywords:** Percutaneous left atrial appendage closure devices, Atrial fibrillation, Embolic stroke, Epicardial ligation

## Abstract

Atrial fibrillation (AF) is the most common arrhythmic disorder world-wide, accounting for 15 % of all strokes. Management of stroke risk in AF is complicated by intolerance of anti-coagulation (AC) therapy and difficulty maintaining therapeutic range in patients treated with warfarin. The left atrial appendage (LAA) is a source of thrombus in AFrelated thrombo-embolic events and surgical LAA exclusion (LAAO) is commonly performed during cardiac surgery in AF patients. Surgical approaches are limited by a high incidence of incomplete closure with a potential for consequent thrombo-embolic events as well as the morbidity of an open-heart procedure. More recently, percutaneous approaches to LAAO have been developed. The LARIAT device is an epicardial LAA exclusion system that enables percutaneous suture ligation of the LAA via combined, pericardial and trans-septal access. The device has 510k Federal Drug Administration (FDA) clearance for soft-tissue ligation and has been studied in canine models in pre-clinical studies as well as published series of clinical experience with LARIAT LAAO. The history, patient selection, procedural technique and complications of LARIAT LAAO are reviewed here. Additionally, insights and procedural improvements that have been elucidated from clinical series and outcomes from the collective experience are discussed. The LARIAT’s epicardial approach to LAA ligation is unique compared with other percutaneous LAA exclusion devices, however more data regarding device safety and efficacy is needed for the LARIAT to emerge as an established therapy for stroke prevention in AF.

## Background

Atrial fibrillation (AF) is the most common arrhythmic disorder worldwide, affecting approximately 2.3 million people in the United States and 4.5 million in the European Union (January et al. 2014). With age, the prevalence and disease burden of AF increases, accounting for 15 % of all strokes and with greater associated morbidity and mortality than non-AF related strokes (January et al. 2014; Connolly et al. 2009). Oral anti-coagulation (AC) is the mainstay of stroke prevention therapy but is complicated by bleeding events and prescribing complexity, with only 50–60 % of patients treated with warfarin consistently in therapeutic range (Go et al. 1999). New oral anti-coagulants (NOACs) such as the direct thrombin inhibitor, dabigatran, and factor Xa inhibitors, rivaroxaban and apixaban, provide consistent AC compared with Coumadin but are limited by bleeding complications, expense and inability to expeditiously reverse these agents during an acute bleeding event (Connolly et al. 2009; Patel et al. 2011; Granger et al. 2011). These challenges have led to a focus on alternate therapies to reduce stroke risk in patients with AF.

The left atrial appendage (LAA) has a narrow-orifice with a tubular, trabeculated structure that fibrillates rather than contracts in AF, resulting in blood stasis and predisposition to thrombus formation (Al-Saady et al. 1999; Kanmanthareddy 2014). In a meta-analysis of 23 studies of AF patients, thrombus, when present, was localized to the LAA in 91 % of patients with non-valvular AF. The implication of the LAA as a primary source of thrombus for thrombo-embolic events in non-valvular AF has made it a veritable target for stroke reduction.

Surgical LAA exclusion by excision or ligation of the LAA, when combined with the Cox-Maze procedure, has demonstrated proficiency in reducing subsequent stroke risk (Bonow et al. 2008; Cox et al. 1999). Notably, a 36 % incidence of incomplete exclusion with surgical ligation alone has been observed and associated with thrombus formation in the partially excluded LAA as well as subsequent thrombo-embolic events (Katz et al. 2000). The flaccid state of the LAA on cardiac bypass and proximity of the circumflex artery to the base of the LAA, have been proposed etiologies of sub-optimal success with surgical approaches. Excision of the LAA provides more consistent results and the 2014 AHA/ACC/HRS Guidelines for the management of atrial fibrillation provide a Class IIB/Level of Evidence C, recommendation for surgical excision of the LAA in patients undergoing cardiac surgery (January et al. 2014). However, surgical excision remains limited by the morbidity of an open-heart procedure and lack of robust efficacy data. Surgical experience has inspired and informed the development of percutaneous left atrial appendage occlusion (LAAO) devices. The LARIAT ligation system is currently the most studied percutaneous epicardial LAAO device.

## LARIAT ligation

### Device development

The LARIAT device was developed by a cardiothoracic surgeon and has United States Federal Drug Administration (US FDA) 510k clearance for the indication of soft-tissue approximation with greater than 2000 implants world-wide for LAA ligation (Price and Gibson 2014). A second generation of the device accommodating larger LAAs is also now commercially available. The LARIAT system accomplishes percutaneous delivery of a suture that snares the LAA epicardially, at its os, via trans-septal and pericardial access. Pre-clinical canine studies demonstrated angiographic LAA exclusion utilizing LARIAT LAAO, confirmed by macroscopic evaluation and showed progressive LAA atrophy and endothelialization of the LAA orifice in a time-dependent manner from ligation (Lee et al. 2010). The utility of an endoluminal, balloon, placed at the os of the LAA to guide LARIAT snare placement and prevent suture slippage was elucidated in an animal trial as well (Singh et al. 2010). Subsequent clinical trials have led to the use of LARIAT ligation most widely for an off-label indication of left atrial appendage ligation for stroke reduction.

### Patient selection

Patient selection (Table 1) for the LARIAT LAAO is guided by the initial safety and feasibility trial completed by Bartus et al. and by experiences published by early operators (Price and Gibson 2014; Stone et al. 2013; Bartus et al. 2013; Massumi et al. 2013). Bartus et al. included: AF patients with a CHADS^2^ score of ≥1 with one of: (1) contraindication to AC, including gastrointestinal, intra-cerebral, urologic or pulmonary bleeding, (2) cerebrovascular accident (CVA) despite adequate AC or (3) indication for ‘triple-therapy’ with aspirin, thienopyridine and AC with high bleeding risk (Bartus et al. 2013).

**Table 1:** LARIAT LAAO patient selection (Bartus et al. 2013)

Clinical inclusion recommendations	Clinical exclusion recommendations	Anatomical exclusion recommendations
Atrial fibrillation with CHADS^2^ score ≥1	History of prior cardiac surgery Myocardial infarction within 3 months	LAA width >40 mm
Contraindication to AC therapy including:	History of pericarditis	Superiorly oriented LAA with the LAA apex directed behind the pulmonary trunk
Gastrointestinal bleeding	History of thoracic radiation	Multi-lobed LAA in which lobes are oriented in different planes exceeding 40 mm
Intra-cranial bleeding	Pectus Excavatum	Posteriorly rotated heart
Urologic bleeding	Thromboembolic event within 1 month	
Pulmonary bleeding	New York Heart Association Class IV heart failure	
Recurrent CVA despite adequate AC therapy	Left ventricular function <30 %	
Requirement for aspirin, thienopyridine therapy and AC therapy with high-bleed risk		
Intolerance to AC therapy		

*INR* international normalized ratio, *CVA* cerebrovascular accident

Contraindications include prior pericarditis or pericardiotomy and thoracic radiation, as pericardial adhesions complicate pericardial access required for LARIAT LAAO. Due to appendage manipulation during the procedure, active thrombus within the LAA is also contraindicated. Additionally, Bartus et al. excluded patients with a myocardial infarction within 3 months, thromboembolic event within 30 days, New York Heart Association (NYHA) Class IV heart failure and left ventricular ejection fraction <30 %.

LARIAT candidates undergo an anatomical evaluation with a cardiac-gated, computed tomography (CT) scan with contrast and 3D image reconstruction to evaluate for anatomical exclusions that preclude successful device advancement. These include: (1) LAA width >40 mm, (2) superiorly oriented LAA with the apex directed behind the pulmonary artery (PA), (3) multi-lobed LAA in which lobes are oriented in different planes exceeding 40 mm, and (4) posteriorly rotated heart.

### Procedure

**Fig. 1:**
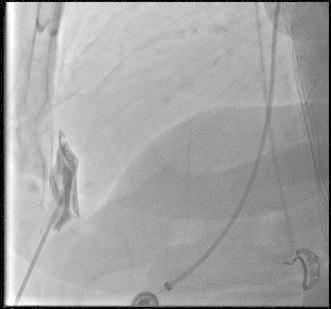
Advancement of the pericardial access needle with small injections of dye allows recognition of pericardial access site with tenting of the pericardium

**Fig. 2:**
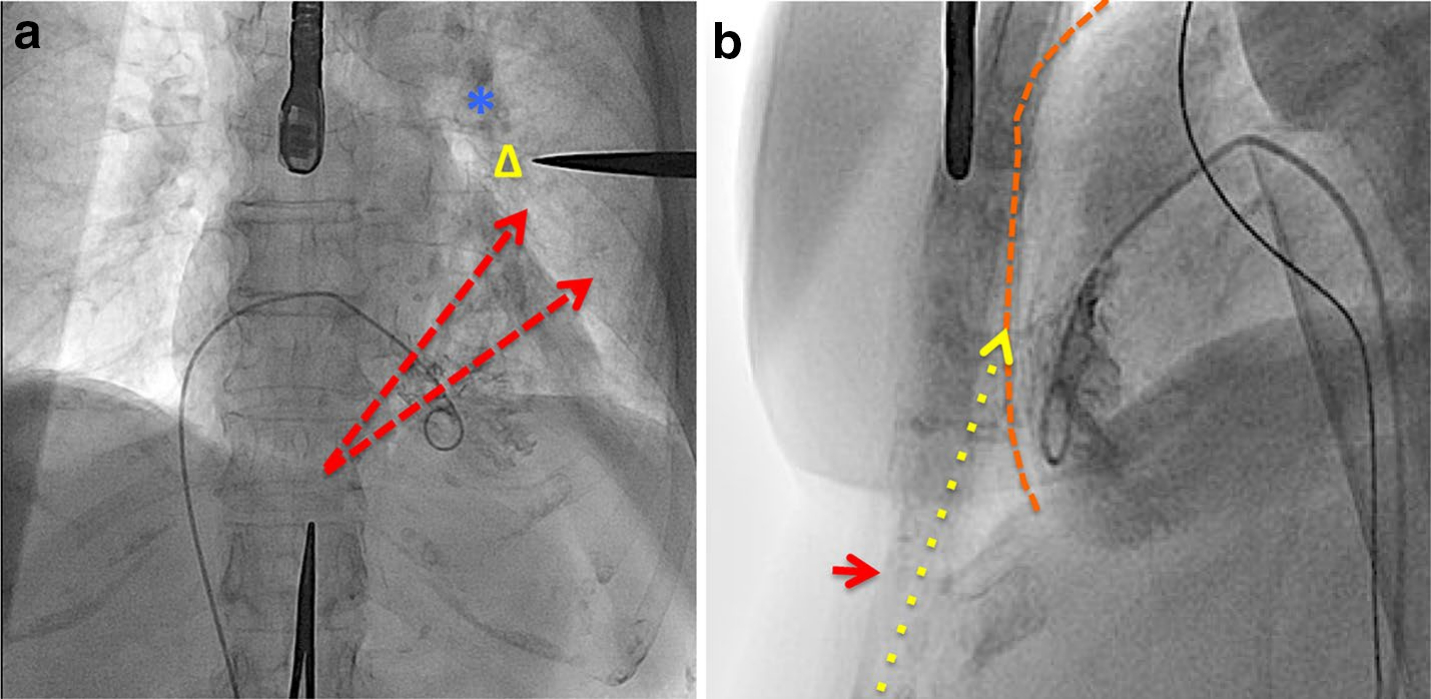
Pericardial access planning in the AP and LL projections. **a** In the AP projection, Kelley clamps are placed below the sub-xiphoid process and at the likely position of the LAA (Δ) based on the position of the PA (*). The ideal trajectory of trans-septal sheath placement is lateral to the LAA in the region between the *dashed red arrows*. **b** In the LL view, the pericardial silhouette (*orange dashed line*) can be approximated by performing a right ventriculogram outlining the RV endocardium. The anterior pericardium is entered following the tract of the *dotted yellow arrow*. The xiphoid process tip is noted at the *short red arrow*

The LARIAT procedure has been extensively detailed as have recommendations to minimize complications and optimize outcomes (Lee et al. 2010; Singh et al. 2010; Bartus et al. 2011, 2013; Valderrabano 2014; Price 2014; Koneru et al. 2014). LARIAT LAAO typically takes place in a cardiac catheterization laboratory (CCL), electrophysiology laboratory (EPL) or hybrid operating room and is implanted by electrophysiologists and interventional cardiologists under general anesthesia with trans-esophageal echocardiography (TEE) guidance. The procedure requires optimal pericardial access described in Figs. 1 and 2 as well as trans-septal access. Ligation occurs with advancement of the LARIAT device via the pericardial sheath over a rail system created by the attached endocardial and epicardial guidewires, followed by snare-capture of the LAA.

A pericardial drain is typically left in place and removed the following day if output is minimal. Patients are discharged 24–48 h after uncomplicated implantation and surveillance TEE is performed at 4–6 weeks post-implant. Pain management approaches include Tylenol as well non-steroidal anti-inflammatory agents. Scheduled colchicine for 2 weeks following LARIAT ligation has been effective in reducing the incidence of post-procedural pericarditis and pain. Anti-platelet and AC regimens following LARIAT ligation in published experience are variable with some patients continued on AC therapy if tolerated, others treated with Aspirin or Plavix or both for a period of time (Koneru et al. 2014).

### Imaging

**Fig. 3:**
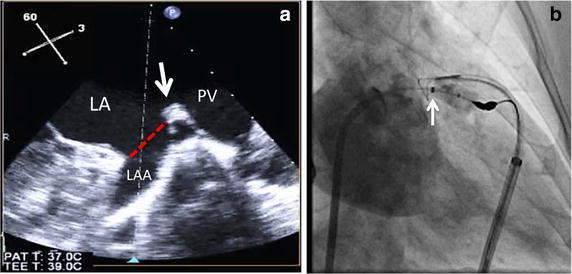
**a** The *dashed line* indicates the desired ligation site, just inferior to the Coumadin ridge (*arrow*). **b** The proximal end of the balloon is positioned at the LAA orifice under TEE guidance. The radio-opaque marker at the proximal end of the balloon (*arrow*) guides advancement of the LARIAT system over the LAA os under fluoroscopy; *PV* pulmonary vein

**Fig. 4:**
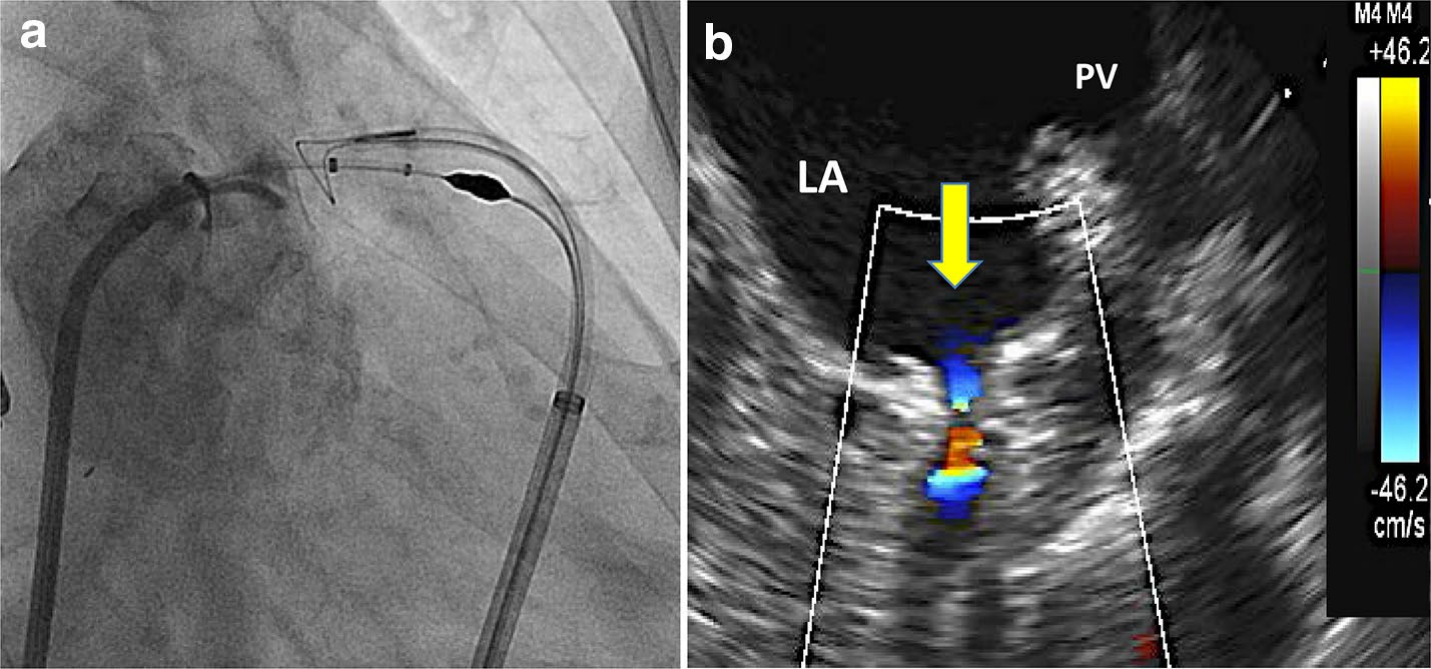
**a** LA angiography post LARIAT LAAO without flow into the snared LAA **b** TEE demonstrating residual 1 mm jet of flow into the ligated LAA (*yellow arrow*)

Laura et al. have detailed the role of multi-modality imaging during LARIAT ligation (Laura et al. 2014). A contrast, cardiac-gated CT is utilized to plan pericardial access and provides information regarding anatomical features such as pulmonary artery enlargement, large xiphoid process or tight retrosternal space and delineates the course of the phrenic nerve and internal mammary artery. CT can also exclude thrombus pre-procedurally as well as indicate the presence of accessory LAAs or diverticula (Ismail et al. 2015). TEE confirms the absence of LAA thrombus, guides placement of the snare at the LAA os by allowing endocath balloon visualization and provides surveillance for pericardial effusion development during the procedure (Fig. 3). TEE and LA angiography are utilized for confirmation of closure, to assess for residual jets immediately post-procedure and to guide further suture tightening (Fig. 4).

### Peri-procedural complications

Complications during LARIAT LAAO can occur during trans-septal, pericardial or venous access as well as during LARIAT delivery. Technical approaches to prevent LARIAT LAAO complications are summarized in Table 2 (Price 2014).

**Table 2:** Potential prevention strategies for procedural complications of LARIAT LAAO (Price 2014; Keating et al. 2014)

Complication	Cause	Preventative strategy
Pericardial effusion	Initial TSP Guide wire or catheter trauma to LAA after TSP Manipulation of delivery system in pericardium Pericardial access	TEE guidance Avoidance of severe IAS tenting Advancement of trans-septal sheath dilator into LAA under fluoroscopy over 0.32″ wire with distal curve on coronary wire TEE surveillance for RV compression with sheath advancement to avoid RV abrasion Micro-puncture access needle Placement of a ‘bail out’ wire in the pericardium for quick pericardial drain placement
LAA laceration or perforation	LARIAT advancement and deployment	Cognizance of endocardial and epicardial wire forces on LAA Minimization of LARIAT delivery system prolapse onto LA Careful suture tightening
Procedural stroke	LAA thrombus Insufficient AC Air embolus	Careful baseline TEE Close AC monitoring Careful flushing of trans-septal sheath
Vascular complications	Hematoma, arterio-venous fistula, pseudoaneurysm, bleeding, hematoma	Careful technique with ultrasound guidance as needed

*TSP* trans-septal puncture, *IAS* inter-atrial septum, *RV* right ventricle

Pericardial effusion and tamponade from RV puncture or abrasion during sheath advancement can complicate pericardial access. Coronary or epigastric artery laceration, trauma to intra-abdominal organs and pleural puncture have also been observed with LARIAT LAAO but should be avoidable with review of pre-procedural CT (Price and Gibson 2014; Stone et al. 2013). Utilizing a micro-puncture needle for pericardial access may mitigate the risk of significant RV laceration. When encountered, adhesions should lead to consideration for aborting ligation given low likelihood of procedural success in this setting and higher risk of complication. Effusions should be promptly treated with drainage and reversal of AC as well as consideration for surgery and can be managed anticipatorily with placement of an extra ‘bail out’ wire in the pericardial space to provide pericardial access for expedited drainage of a large effusion (Price 2014). Late pericardial effusions may develop and are hypothesized to result from inflammation related to LAA necrosis. Late pleural effusions have also been noted and may be transudative or exudative and potentially represent volume retention from reduced atrial natriuretic peptide (ANP) release after LAA ligation (Gunda et al. 2015).

Traction forces on the LA during LARIAT advancement and suture tightening can lead to LAA laceration or perforation and need for surgical rescue. Keating et al. reported LA laceration and cardiac tamponade requiring surgical intervention in 3 of 6 LARIAT ligations performed at their center (Keating et al. 2014). Reducing catheter prolapse onto the LA, particularly when the LA is enlarged, is a recommended preventative practice for LA or LAA laceration. Deployment of the LARIAT at a position with sufficient laxity such that the appendage orifice is recreated by proximal LAA tissue results in lesser traction on neighboring LA tissue. LAA perforation can occur during connection of the endo- and epi- wires, when tension imposed on the friable LAA can cause the epicardial wire to perforate. While prompt LARIAT deployment is a definitive treatment for LAA laceration or perforation, surgical readiness and a low threshold for surgical evaluation of ongoing pericardial output is recommended to avoid rapid decompensation.

## Clinical experience and outcomes

Results of the published clinical experience with the LARIAT device with greater than ten patients are reviewed in Tables 3, 4 and cumulative event rates of series only closed-chest ligation are summarized in Table 5.

**Table 3:** Review of patient characteristics in published series of greater than ten patients of LARIAT LAAO

Referenes	No. of patients with attempted ligation	No. of patients meeting clinical criteria screened	Mean age	Male (%)	AC Post-procedure	Mean/median CHADS^2^ Score/CHA_2_DS-VASC Score^a^ (Lip et al. 2010)	HAS-BLED Score (Lip et al. 2011)
Bartus et al. (2011)	12	14 screened, 2 excluded: 1 due to sub-optimal anatomy by pre-procedure CT, 1 due to presence of LAA thrombus by TEE at procedure onset	57.3	62	NR	NR	NR
Bartus et al. (2013)	92	119 screened, 27 excluded: 16 due to sub-optimal anatomy by pre-procedure CT, 11 due to mobile thrombus noted by TEE at time of procedure	62 ± 10	57	Warfarin if tolerated, else aspirin mono-therapy. 55 % treated with warfarin post-procedure	1.9 ± 0.95/2.8 ± 1.56	2.4 ± 1.1
Massumi et al. (2013)	20	NR	73 ± 8	65	65 % continued on Aspirin, 20 % on clopidogrel, 5 % on dual-antiplatelet therapy with Aspirin and dypyridamole, 15 % on warfarin, 5 % on rivaroxaban, 20 % on no AC	3.2 ± 1.2/4.8 ± 1.3	3.5 ± 1.0
Stone et al. (2013)	27	42 screened, 15 excluded; no further details	75 ± 8	74	Daily aspirin in all patients, dual anti-platelet therapy in 9 patients	3.5 ± 1.4/5.1 ± 1.5	4.6 ± 0.9
Price and Gibson (2014)	154	NR	72 ± 9	62	Aspirin mono-therapy 31 %, dual anti-platelet 24 %, oral AC 23 %, clopidogrel mono-therapy 7 %, aggrenox 0.6 %	2.8 ± 1.4/4.1 ± 1.6	3.2 ± 1.2
Miller et al. (2014)	41	NR	75 ± 10	46	At last follow-up, Aspirin 46 %, warfarin 20 %, Plavix 7 % dabigatran 7 %, rivaroxaban 7 %	3.0 ± 1.3	4.4 ± 1.4

*AC* anti-coagulation, *CT* computed tomography scan, *LAA* left atrial appendage, *TEE* trans-esophageal echocardiography, *NR* not reported

^a^Medians are presented with an interval, means are presented with a standard deviation

**Table 4:** Review of device success and peri-procedural and late complications from published clinical experience with percutaneously or minimally-invasively delivered LARIAT suture delivery device with greater than ten patients

Study	Device success defined by <5 mm leak	Causes of failure to complete ligation	Durable ligation by follow-up TEE defined by <5 mm leak	Peri-procedural complications	Late complications	Median or mean procedural time (min)^a^	Hospital LOS (days)
Bartus et al. (2011)	83 % (10/12)	1 failure to complete ligation due to inadequate TEE guidance, 1 pericardial adhesion preventing access	6/6 patients undergoing 60 days follow-up TEE had durable ligation	1 patient with pectus excavatum required thoracotomy for device removal	NR	85.7 [22–335]	NR
Bartus et al. (2013)	92 % (85/92)	3 pericardial adhesions preventing access, 1 pericardial adhesion preventing device advancement, 2 peri-procedural complications requiring termination, 1 anatomical contraindication to trans-septal puncture	85/85 patients undergoing 30 days TEE follow-up had durable ligation while 65/65 patients undergoing 1 yr TEE follow-up had durable ligation	1 epigastric artery laceration requiring cauterization, 1 RV puncture requiring pericardial drainage, 1 perforation during trans-septal access requiring pericardial drainage, 1 adhesion preventing advancement of LARIAT device, 3 adhesions preventing access, 2 severe pericarditis	2 non-embolic CVA, 2 SCD remote from procedure, 1 late effusion, 1 LA thrombus noted at 1 yr follow-up TEE resolving with warfarin therapy	45 [36–55]	NR
Massumi et al. (2013)	100 % (20/20)	None	17/17 patients undergoing follow-up TEE at a mean of 96 days had durable ligation. In 6/17 patients, a residual pouch was noted with smooth walls in 5 and few pectinate muscles in 1	1 RV puncture requiring surgical intervention, 1 cardiac tamponade requiring pericardiocentesis, 1 prolonged intubation, 3 pericarditis with 1 requiring repeat pericardiocentesis	3 pericarditis, 1 death due to sepsis and pulmonary embolism occurring 50 days after ligation thought un-related to the procedure	83 ± 21	3.7 ± 3
Stone et al. (2013)	93 % (25/27)	2 peri-procedural complication requiring termination	22/22 patients undergoing TEE follow-up at a mean of 40 days had durable ligation	1 LAA laceration treated with reversal of anti-coagulation followed by surgical MAZE and appendage ligation, 1 CVA attributed to trans-septal sheath thrombus occurring in setting of sub-therapeutic ACT with no major neurologic sequelae after neurovascular rescue, 3 pericarditis	1 CVA 33 days post-procedure, thought secondary to arch atheroma, 1 pleural effusion	73 ± 18	2.8 ± 1.6
Price and Gibson (2014)	94 % 144/154	2 pericardial adhesions preventing access, 2 pericardial adhesions preventing device advancement, 2 difficult anatomy precluding ligation, 2 peri-procedural complications requiring termination	59/63 patients undergoing follow-up TEE had durable ligation with 4 having a >4 mm leak. Thrombus in the LA was noted in 3 patients undergoing TEE and 1 patient undergoing CT	3 patients required surgical exploration (2 for RV puncture, 1 for LAA perforation), 1 patient death due to nosocomial pneumonia post-procedure, 16 pericardial effusions, 4 pleural effusions	At a mean of 112 days follow-up, 2 cardiovascular deaths, 1 non-cardiovascular death, 2 CVAs, 3 pericardial effusions, 3 pleural effusions, 4 patients with thrombus noted in LA by TEE or CT	NR	NR
Miller et al. (2014)	95 % (39/41)	2 peri-procedure LAA perforation requiring emergent surgery	39/39 patients undergoing follow-up TEE had durable ligation	4 LAA lacerations (2 required exploratory surgery, 1 managed with pericardiocentesis, 1 managed with ligation), 13 pericardial effusions, 7 pericarditis, 4 pleural effusions	1 CVA, 5 pericardial effusions, 2 pericarditis, 2 pleural effusions	127 ± 50	NR

*TEE* trans-esophageal echocardiography, *LOS* length of stay, *RV* right ventricle, *CVA* cerebrovascular accident, *SCD* sudden cardiac death, *LA* left atrium, *LAA* left atrial appendage, *NR* not reported

^a^Median times are presented with an interval, mean times are presented with a standard deviation

**Table 5:** LARIAT LAAO success and durability and procedural and late adverse events from published series with greater than 10 patients and closed-chest ligation^1^ (Price and Gibson 2014; Stone et al. 2013; Bartus et al. 2013; Massumi et al. 2013; Miller et al. 2014)

	Number of patients
Device success^a^	313/334 (94 %)
Device durability^b^	222/226 (98 %)
Procedural adverse events^c^	64/334 (14.7 %)
Death	1/334 (0.3 %)
LAA laceration	6/334 (1.8 %)
CVA/TIA	1/334 (0.3 %)
Significant pericardial effusion^d^	25/334 (7.5 %)
Complication with surgical intervention	8/334 (2.4 %)
Pericarditis^e^	15/180 (8.3 %)
Pleural effusion	8/334 (2.4)
Late adverse events	33/334 (9.9 %)
Death	6/334 (1.8 %)
CVA/TIA	6/334 (1.8 %)
Pleural effusion	6/334 (1.8 %)
Pericardial effusion	10/334 (3.0 %)
Thrombus in LA or LAA by TEE/CT	5/227 (2.2 %)

^a^Successful deployment of device with <5 mm leak by TEE/CT

^b^LAA leak <5 mm by last follow-up TEE/CT in those whom follow-up imaging available

^c^Events occurring prior to discharge and not including pericarditis

^d^Effusions requiring pericardiocentesis or vasopressor therapy

^e^Price et al. did not provide pericarditis rate

The first-in-man feasibility study of the LARIAT device evaluated 13 patients undergoing LARIAT ligation either during open-heart surgery or in a closed-chest fashion. Twelve of 13 patients in this series had successful LAA ligation with 1 patient in whom the procedure was terminated due to lack of adequate echocardiographic guidance for snare advancement. Notably, a patient with pectus excavatum required a thoracoscopic procedure for device removal due to sternal compression (Bartus et al. 2011).

Bartus et al. subsequently published experience with LARIAT ligation in 92 patients from a single center, where ligation was successfully completed in 85 of 92 or 93 % of subjects. At 1-year follow-up, 65 patients underwent follow-up TEE with all patients having <5 mm leak. Notably, 55 % of the patients in this series were continued on AC therapy (Bartus et al. 2013).

Massumi et al. reported the first series of LARIAT ligation performed in the (US) in a single-center report of 20 patients. All attempted ligations were successful however peri-procedurally, 1 patient required surgical intervention for RV perforation and 1 patient was treated with pericardiocentesis for tamponade physiology. All 17 patients undergoing follow-up TEE at a mean of 96 ± 77 days had persistent LAA occlusion. However, in 6 of 17 patients, a small pouch was noted at the LAA os, containing smooth muscle tissue in 5 patients and pectinate muscles in one patient. Involution of the excluded LAA was noted in 3 patients in whom follow-up CT imaging was performed (Massumi et al. 2013).

Stone et al. reported a series of 27 US patients, selected from 42 patients being evaluated for LAAO that underwent LARIAT ligation, with 25 of 27 having successful ligation. One peri-procedure stroke was attributed to a sub-therapeutic ACT with thrombus noted on the trans-septal sheath. All 22 patients completing follow-up TEE at a mean of 45 days had durable ligation (Stone et al. 2013).

The largest US LARIAT experience studied 154 consecutive patients undergoing LAA ligation at eight centers. Device success, defined as device deployment with <5 mm residual leak by TEE, was achieved in 94 % of patients. Major bleeding occurred in 9 % of patients, and peri-procedural pericardial effusion in 16 %. Despite similar device success rates as other series, a higher rate of late-leak (20 %) and LA thrombus (4.8 %) was noted in follow-up. Of note, the patients included in this study were older and had more co-morbidities than the initial single center study of Bartus et al. (Price et al. 2014).

Miller et al. reported on an additional 41 consecutive patients undergoing LARIAT ligation with 39/41 having procedural success. A high rate of pericardial effusions requiring pericardiocentesis post-procedurally was noted (20 %), which authors attributed to operators in this series not maintaining a pericardial drain post-procedurally. Seven percent of patients required thoracentesis for late pleural effusions. Authors also noted a high rate of LAA perforation, with 2 of 4 patients with this complication requiring surgical treatment. All 4 patients with LAA perforation required multiple attempts to position the LARIAT snare, suggesting that in cases of challenging anatomy, advancement should be attempted when endocardial and epicardial wire alignment is optimal and aborted after a limited number of attempts to avert laceration and perforation (Miller et al. 2014).

Gafoor and authors evaluated the safety and efficacy of LAAO with a number of occlusion devices in a cohort of 75 octogenarians. Procedural success was noted in all 4 patients undergoing LARIAT exclusion, with no acute adverse safety events and an average hospital length of stay of 2.5 days. At 1-year, 1 LARIAT patient had an embolic stroke with thrombus originating from an incompletely ligated lobe of the appendage (Gafoor et al. 2014).

Patel et al. evaluated the compassionate use of LARIAT ligation in 9 patients who were precluded based on appendage morphology and size. Their analysis showed a LARIAT deployment success rate of 78 % utilizing strategies such as using the magnet-tipped endowire to straighten the LAA to reduce circumference and utilizing the endocath balloon to suction from the LAA, effectively reducing LAA volume (Patel et al. 2015).

**Fig. 5:**
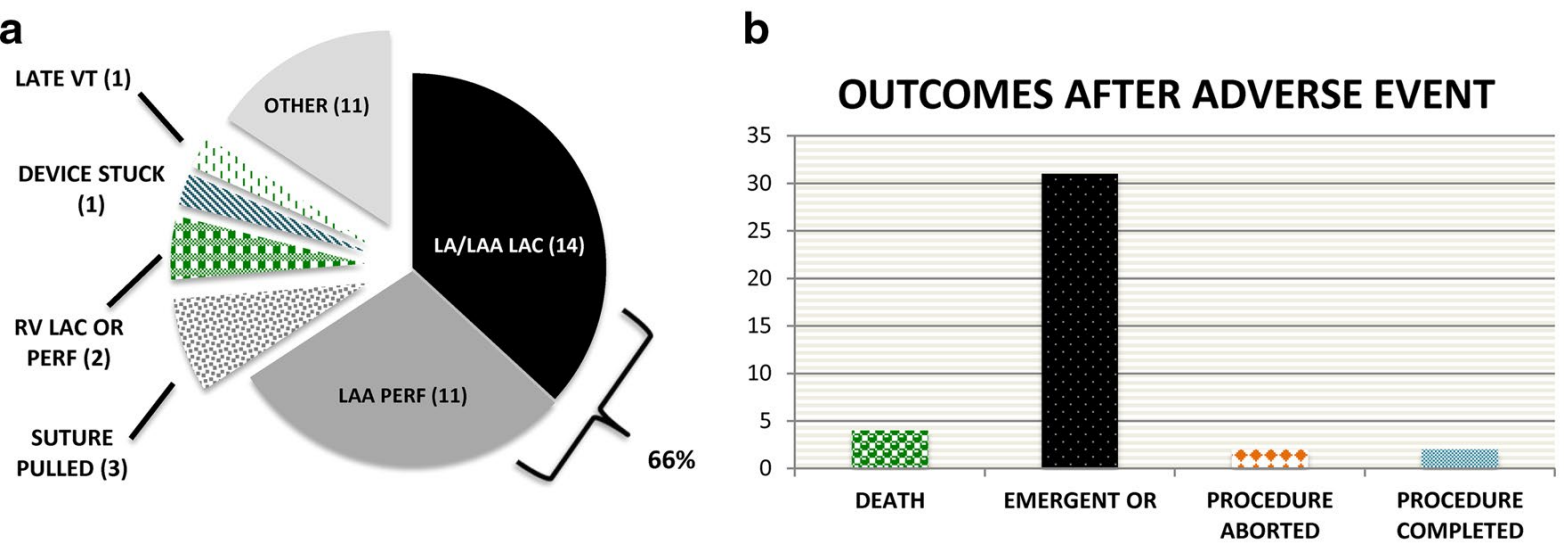
FDA MAUDE database review. **a** Types and frequencies of reported adverse events. **b** Outcomes after an adverse event; *VT* ventricular tachycardia, *LAC* laceration, *PERF* perforation, *OR* operating room

The FDA’s Manufacturer and User Facility Device Experience (MAUDE) reports 38 LARIAT-related adverse events from January 2012 to March 2015, including 31 instances requiring emergent sternotomy for bleeding complications following LARIAT attempts, 4 deaths and 1 episode of unexplained VT linked to myocardial scar after LARIAT ligation (Fig. 5). LA/LAA laceration or perforation accounted for 66 % of reported events, with LA/LAA laceration most commonly resulting from multiple attempts at advancing the suture delivery system over the LAA while perforation most commonly resulted from endowire trauma.

## Discussion

Several features distinguish the LARIAT LAAO system. Compared with percutaneous endocardial LAAO devices such as the WATCHMAN (Boston Scientific Corporation, Natick, Massachusetts) and Amplatzer (St. Jude Medical, Inc., Saint Paul, Minnesota), systems, the LARIAT approach is epicardial, with only a polyester suture left behind. Device embolization, noted in 1.2 % of patients in the PREVAIL trial of the WATCHMAN device, is not observed with LARIAT LAAO and late device erosion is not a concern (Holmes et al. 2014). Intriguingly, involution of the LAA has been noted on CT imaging following LARIAT ligation and as early as 4 weeks post-implant on autopsy findings, which may have a desirable impact on the long-term durability of this approach (Massumi et al. 2013; Ellis et al. 2015). LARIAT LAAO results in electrical isolation of the LAA, with post-procedural reduction in AF burden noted as well as increased maintenance of NSR observed when performed in conjunction with pulmonary vein isolation (Han et al. 2014; Afzal et al. 2015; Badhwar et al. 2015). In the PROTECT-AF and CAP registries of the WATCHMAN device, a less than 5 mm peri-device residual leak into the appendage defined procedural success, whereas several LARIAT series demonstrate no or minimal residual jet with a similar rate of procedural success (Bartus et al. 2013; Massumi et al. 2013; Miller et al. 2014; Holmes et al. 2009; Reddy et al. 2011). In case series, 6.25–35.7 % of patients screened for LARIAT LAAO are excluded due to anatomical exclusions, while in the PROTECT-AF experience of the WATCHMAN device, 38.9 % of patients screened for device placement were excluded for clinical and echocardiographic reasons, suggesting that a complement of LAAO approaches may be suitable to serve a clinically an anatomically diverse population of AF patients (Stone et al. 2013; Bartus et al. 2011, 2013; Holmes et al. 2009).

**Fig. 6:**
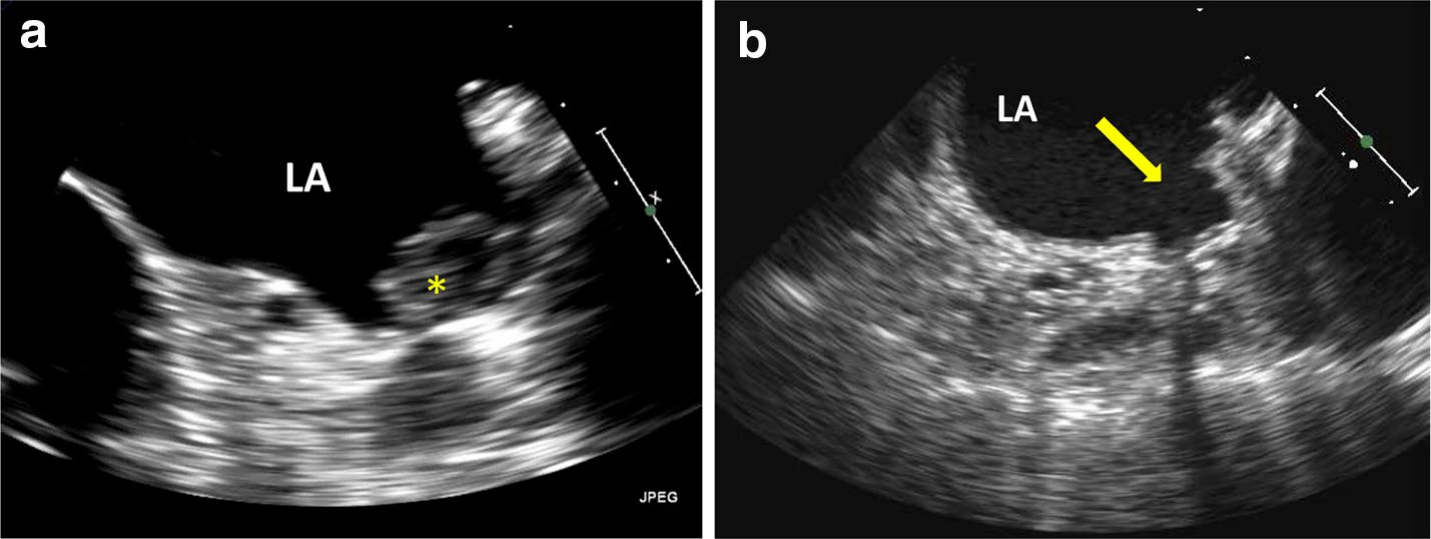
Complications noted during follow-up TEE after LARIAT LAAO. **a** Thrombus at the site of LAA ligation (*). **b** Residual LAA stump with pectinate muscle in LA (*arrow*)

Widespread adoption of LARIAT LAAO is limited by lack of efficacy data, with utilization rationalized by presumed efficacy extrapolated from other appendage exclusion mechanisms, and minimal data regarding rate of long-term thrombotic events and procedural complications. Theoretically, AC after LARIAT LAAO is not required as only an epicardial suture is retained, however in the cumulative published experience, a TIA/CVA was observed in 1.8 % of patients in follow-up (Table 5) and AC or anti-platelet therapy was continued in 55 % of patients in the controlled Bartus et al. series, reflecting uncertainty regarding residual thrombo-embolic risk in the absence of randomized trial data (Bartus et al. 2013). Comparatively, AC therapy was discontinued in most patients 45 days after WATCHMAN implant in the PREVAIL and PROTECT-AF trials (Holmes et al. 2009, 2014). Several case reports detail thrombus at the site of the LAA orifice on surveillance imaging following LARIAT LAAO and thrombus was noted in 2.2 % of cases in the cumulative published experience (Table 3; Fig. 6a) (Price and Gibson 2014; Bartus et al. 2013; Briceno et al. 2013; Koranne et al. 2015; Giedrimas et al. 2013; Baker et al. 2013). Thrombus formation results from an inflammatory environment at the ligation site, epithelial denuding at the LAA orifice during balloon catheter retrieval and sub-optimal suture deployment with a remnant thrombus-promoting static LAA chamber (Fig. 6b) (Bartus et al. 2014). Additionally, recurrent LA-LAA communication after initial successful LARIAT LAAO can result from knot-loosening and tissue necrosis at the suture site and is another mechanism of thrombo-embolic complication. In case reports, late leaks have been treated successfully with alternate LAAO devices as repeat LARIAT ligation is not typically pursued due to potential pericardial adhesions developed after the initial procedure (Yeow et al. 2013; Mosley et al. 2014; Di Biase et al. 2013; Pillai et al. 2014). The implications of residual leak are unknown, however in a review of 259 patients who underwent LARIAT ligation, 14 % were noted to have recurrent LA-LAA communication at 1 year, compared with 21 % in patients undergoing WATCHMAN endocardial occlusion. Most commonly recurrent communication was of a central or ‘gunny sack’ pattern, with no link with CVA observed (Pillarisetti et al. 2015). Additionally, in a post hoc and underpowered analysis of the PROTECT-AF trial, increased thrombo-embolic events were not noted in follow-up of patients with persistent peri-device flow with the WATCHMAN device (Viles-Gonzalez et al. 2012).

The incidence of peri-procedural complications during LARIAT LAAO is poorly delineated and derived from one controlled trial evaluating the device in 92 subjects and experience from reported case series. A peri-procedural death rate of 0.3 % is noted in the cumulative published LARIAT experience, however the FDA’s MAUDE database reports four procedure-related deaths, likely reflecting publication bias present in reported case-series (Table 5; Fig. 5). The emergent surgery rate in the cumulative LARIAT experience was 2.4 % (8 patients) compared with 31 reports of emergent surgery in the MAUDE database. Notably, the emergency surgery rate was 1.6 % in the PROTECT-AF trial and 0.4 % in the PREVAIL trial (Holmes et al. 2009, 2014). Uniquely, LARIAT LAAO results in a high rate of pericarditis due to pericardial manipulations and appendage necrosis and is also linked to the development of pleural effusions. Long-term implications of pericardial manipulation, inflammation and adhesion formation after LARIAT LAAO are not known. Paucity of data regarding real-world rate of procedural complications and the device’s exclusively off-label use were critiqued in a recent JAMA review (Holmes et al. 2009; Chatterjee et al. 2015). The device is currently being studied in a multi-center observational trial evaluating procedural complication rate and short-term durability [ClinicalTrials.gov, NCT02059707].

A large, controlled trial of LARIAT LAAO would inform the incidence and mechanisms of various complications of LARIAT LAAO as well as possible means of improving upon these in a systematic fashion. Analysis of the PROTECT-AF trial and Continuing Access Registry of the WATCHMAN device allowed for advancements in operator training as well as device refinements and protocol modifications that resulted in significant improvement in the safety and efficacy of the device (Holmes et al. 2009; Reddy et al. 2013). Further insight into anatomical considerations that would enhance current inclusion and exclusion criteria for LARIAT LAAO could also be obtained by a large-scale trial of the device. Delineation of an optimal AC regimen would inform and standardize post-procedural practice. Establishment of efficacy in reduction of thrombo-embolic events compared with Coumadin or NOACs is an important aim for future investigations and for meaningful comparison with other LAAO systems.

## Conclusion

The LARIAT device is an epicardial approach to LAA ligation, with safety and efficacy studied in small clinical series. Epicardial ligation may have potential advantages over endocardial occlusion such as LAA involution and electrical isolation. True complications rates and procedural strategies to prevent and manage complications, efficacy in reduction of thrombo-embolic events, optimal patient selection and post-procedural AC regimens remain to be delineated for the LARIAT ligation system.
